# Campylobacter molothri sp. nov. isolated from wild birds

**DOI:** 10.1099/ijsem.0.006635

**Published:** 2025-02-06

**Authors:** William G. Miller, Bruno S. Lopes, Mary H. Chapman, Tina G. Williams, Meenakshi Ramjee, Delilah F. Wood, James L. Bono, Ken J. Forbes

**Affiliations:** 1 Produce Safety and Microbiology Research Unit, Agricultural Research Service, U.S. Department of Agriculture, Albany, CA, USA; 2 School of Health and Life Sciences, Teesside University, Middlesbrough, UK; 3 National Horizons Centre, Teesside University, Darlington, UK; 4 Bioproducts Research Unit, Agricultural Research Service, U.S. Department of Agriculture, Albany, CA, USA; 5 Wolfson Wohl Cancer Research Centre, Glasgow. The University of Glasgow, Glasgow, UK; 6 Meat Safety and Quality Research Unit, Agricultural Research Service, U.S. Department of Agriculture, Clay Center, NE, USA; 7 School of Medicine, Medical Sciences and Nutrition, University of Aberdeen, Aberdeen, UK

**Keywords:** blackbird, brown-headed cowbird, *Campylobacter*, hippuricase, novel species

## Abstract

Twenty-nine hippuricase-positive *Campylobacter* strains were isolated from wild birds and river water. Previous characterization using *atpA* typing indicated that these strains were related to *Campylobacter jejuni* and *Campylobacter coli* but were most similar to three recently described hippuricase-positive *Campylobacter* species recovered from zebra finches, i.e. *C. aviculae*, *C. estrildidarum* and * C. taeniopygiae*. Phylogenetic analyses using 330 core genes placed the 29 strains into a clade well separated from the other *Campylobacter* taxa, indicating that these strains represent a novel *Campylobacter* species. Pairwise digital DNA–DNA hybridization and average nucleotide identity values were below 70 and 95 %, respectively, thus providing further supporting evidence of a novel taxon. Standard phenotypic testing was performed. All strains are microaerobic or anaerobic, motile, Gram-negative, spiral cells that are oxidase, catalase and nitrate reductase positive, but urease negative. Genomic analyses indicate that the 29 strains can potentially synthesize very few amino acids *de novo* and are auxotrophic for many amino acids and cofactors, similar to the species composing the *Campylobacter lari* group. In addition, these strains encode complete Entner–Doudoroff and Leloir pathways, suggesting that they may possess the ability to utilize both glucose and galactose; these pathways were also identified in the genomes of the zebra finch-associated taxa. The data presented here show that these strains represent a novel species within *Campylobacter*, for which the name *Campylobacter molothri* sp. nov. (type strain RM10537^T^=LMG 32306^T^=CCUG 75331^T^) is proposed.

## Data Summary

One supplementary figure, seven supplementary tables and one supplementary file are provided in the online version of this article. All supplementary data are available through Figshare at https://doi.org/10.6084/m9.figshare.27616641.v1.

The association of *Campylobacter* with domesticated birds, such as poultry, is well established [1,3]. However, campylobacters are also commonly recovered from wild birds [3,7]. *Campylobacter* prevalence was observed to differ between the avian ecological guilds [7], with high, moderate and low prevalence observed in shoreline-foraging birds, ground-foraging insectivores and ground-foraging granivores, respectively. A common feature of many avian-associated campylobacters is the ability to hydrolyse hippuric acid. Hippuric acid (*N*-benzoylglycine) is cleaved by the HipO hippuricase in *Campylobacter jejuni* [8], and the subsequent production of glycine upon cleavage is readily detected using a simple colourimetric assay [9]. Initially, hippuricase activity was primarily associated with *C. jejuni*; although some *C. jejuni* strains did not hydrolyse hippuric acid, the presence of hippuricase activity was considered diagnostic for that species [10]. However, other hippuricase-positive *Campylobacter* species have been subsequently described. These include *Campylobacter avium* recovered from poultry [11], *Campylobacter bilis* and *Campylobacter hepaticus* recovered from chickens [12,13] and *Campylobacter aviculae*, *Campylobacter estrildidarum* and *Campylobacter taeniopygiae* recovered from zebra finches [14]. In addition, *Campylobacter canadensis*, recovered from whooping cranes and originally described as hippuricase-negative [15], also possesses hippuricase activity (data not shown). Most of the novel hippuricase-positive *Campylobacter* species encode HipO [14]; the genome of the *C. canadensis* type strain possesses two *hipO* genes (data not shown). However, *C. avium* does not contain *hipO* but encodes an alternate peptidase M20 domain-containing zinc-dependent aminoacylase/carboxypeptidase termed HipA [16].

**Fig. 1.**
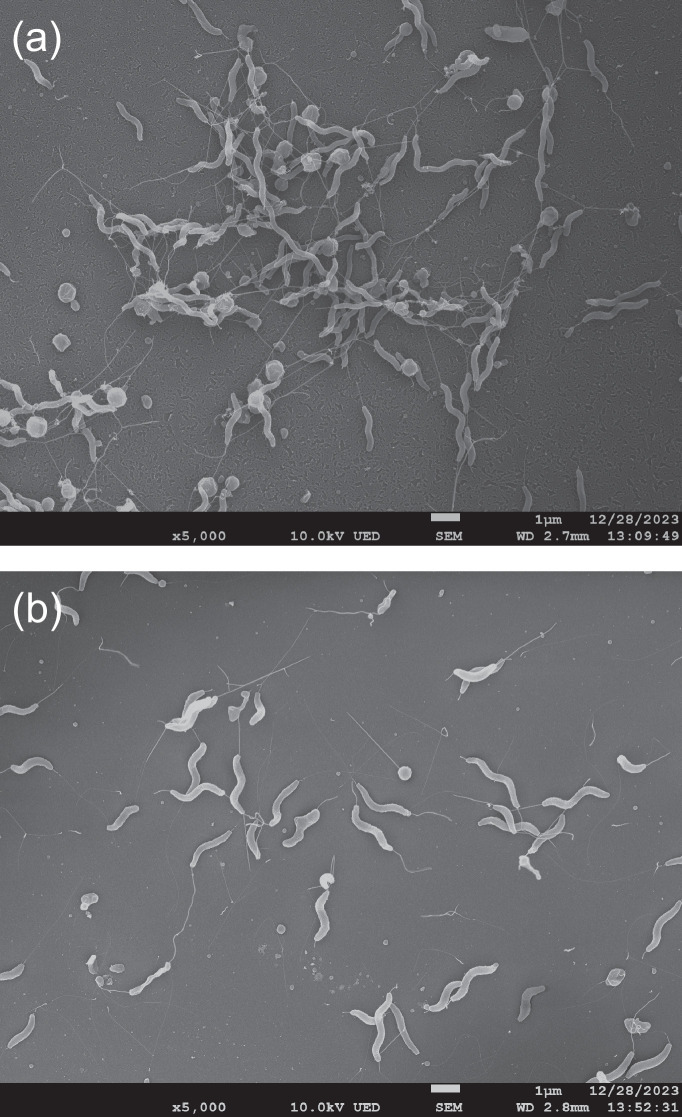
Scanning electron micrograph images of *Campylobacter molothri* sp. nov. (**a**) *Campylobacter molothri* sp. nov. RM10537^T^ (=LMG 32306^T^) and (**b**) *Campylobacter molothri* sp. nov. RM12397 (=LMG 32307) at ×5000 magnification.

Over the course of a multi-year survey within Central California [17], 20 hippuricase-positive *Campylobacter* were isolated from wild birds, primarily brown-headed cowbirds and blackbirds (see Table S1, available in the online Supplementary Material). Sampling was performed as described earlier [17]. Faecal and cloacal swabs were streaked onto anaerobe basal agar plates (ABA; Oxoid, Thermo Fisher Scientific, Waltham, MA) amended with 5% lysed horse blood (Innovative Research, Novi, MI) and CAT supplement [cefoperazone (8 mg l^−1^), amphotericin B (10 mg l^−1^) and teicoplanin (4 mg l^−1^); Oxoid]. Inoculated ABA-blood/CAT plates were grown microaerobically (1–2% O_2,_ 10% CO_2_, 10% H_2 _and ~80% N_2_) for 24–48 h at 37 °C. Samples from positive plates with bacterial growth were examined microscopically for cells with curved or spiral morphology typical of many *Campylobacter* taxa. Cells from these potential *Campylobacter*-containing plates were resuspended in PBS and then filtered through 0.6 µm mixed-cellulose filters (Whatman, Thermo Fisher) [18] onto fresh ABA plates. Growth under the filters was then streaked onto new ABA plates to obtain single colonies. Both sets of ABA plates were incubated microaerobically for 24 h at 37 °C, as described above. All 20 strains were Gram-negative and motile. The cellular morphology of two representative strains, RM10537^T^ and RM12397, was characterized further using scanning electron microscopy. Bacterial samples were prepared for SEM examination as described earlier [19]. Cells from both strains were spiral shaped with bipolar flagella on both ends, and some coccoid cells were also observed (Fig. 1).

Initial *C. jejuni*/*C. coli* antibody tests and 16S rRNA gene sequencing analyses suggested that these 20 strains were related to *C. coli*. However, the strains were all strongly hippuricase-positive, a phenotype not associated with *C. coli*. At the time of isolation, the only two described hippuricase-positive *Campylobacter* taxa were *C. jejuni* and *C. avium*. The atpAF/atpAR primer pair from Miller *et al*. [20] amplified DNA from these strains, indicating that they were not *C. avium*, which does not amplify with this primer pair [21]. Multilocus sequence typing (MLST) of these isolates, using primers designed to amplify multiple *Campylobacter* species, including *C. jejuni*, could not be performed due to inconsistent amplification. These results suggested that the hippuricase-positive isolates were either highly divergent *C. jejuni* or members of a novel hippuricase-positive *Campylobacter* taxon. The latter possibility was further supported by typing 19 of these isolates using more universal *atpA* primers [22]; *atpA* sequences from these isolates formed a clade distinct from other *Campylobacter* species, including *C. jejuni*. Subsequently, nine additional strains were also determined to be the members of this novel cluster, thus yielding a final total of 29 related hippuricase-positive isolates (Table S1). These additional strains included six isolates from a bird ringing study in Scotland, two isolates recovered in 2002 from Sonoma Creek and one isolate recovered from the Salinas River in 2014. For the river/creek samples, 1 ml of water was added to 6 ml 1× anaerobe basal broth (Oxoid) + Preston supplement (10 mg l^−1^ amphotericin B, 10 mg l^−1^ rifampicin, 5 IU ml^−1^ polymyxin B and 10 mg l^−1^ trimethoprim lactate; Oxoid) in a six-well microtitre plate. Plates were placed inside plastic Ziploc freezer bags and enriched microaerobically as above for 24 h at 37 °C and 40 rpm. After incubation, a 10 µl loop of enriched sample was streaked onto an ABA-blood/CAT plate to obtain single colonies. Comparison of the 29 isolates with the hippuricase-positive *C. bilis*, *C. hepaticus*, *C. aviculae*, *C. estrildidarum* and *C. taeniopygiae* using *atpA* typing indicated that they are related to but distinct from the three zebra finch-associated species (data not shown). Since the prospective type strain (RM10537^T^) was recovered from a brown-headed cowbird (*Molothrus ater*), the 29 strains were termed *Campylobacter molothri* sp. nov.

The 29 hippuricase-positive *C. molothri* strains were characterized using standard phenotypic tests, as previously described ([23], see also File S1). Strains were tested for motility; growth at 30, 37 and 42 °C under microaerobic conditions and at 37 °C under aerobic and anaerobic conditions; oxidase, catalase, nitrate reductase, alkaline phosphatase and urease activity; the ability to reduce indoxyl acetate, selenite and triphenyltetrazolium chloride (TTC); α-haemolysis on ABA-blood agar; H_2_S production on triple sugar iron (TSI) media; growth on modified charcoal–cefoperazone–deoxycholate agar (mCCDA) plates or media amended with 1% (w/v) glycine, 0.04% (w/v) TTC, 0.032% (w/v) methyl orange or 2% (w/v) NaCl; anaerobic growth on 0.1% (w/v) trimethylamine *N*-oxide (TMAO) and resistance to 30 mg l^−1^ cephalothin or 30 mg l^−1^ nalidixic acid. All tests were performed three times using appropriate positive and negative controls. The results are presented in Table 1. The phenotypic profile of *C. molothri* was compared to the profiles of other motile, hippuricase-positive *Campylobacter* species (Table 1; a complete comparison to all *Campylobacter* taxa is presented in Table S2). The *C. molothri* strains were unable to hydrolyse indoxyl acetate and were thus distinguishable from *C. jejuni*, *C. avium*, *C. canadensis*, *C. bilis* and *C. hepaticus* (Table 1). However, the phenotypic profile of *C. molothri* is similar, but not identical to those of *C. aviculae*, *C. estrildidarum* and especially *C. taeniopygiae* (Table 1). These phenotypic results are perhaps unsurprising, given the similar avian sources of these taxa. Nevertheless, catalase activity separates *C. molothri* and *C. aviculae*, and 79% of the *C. molothri* strains demonstrate alkaline phosphatase activity, a phenotype not found within the finch-associated taxa.

**Table 1.** Phenotypic characteristics that differentiate *Campylobacter molothri* sp. nov. from the other motile, hippuricase-positive taxa of the genus *Campylobacter*

		1	2	3	4	5	6	7	8	9	10
Temperature (atmosphere)											
	30 °C (microaerobic)	−	−	−	−	−	−	U	U	−	M
	42 °C (microaerobic)	+	+	+	+	+	+	+	+	−	+
	37 °C (anaerobic)	+	+	+	+	−	+	−	−	−	−
Catalase		+	+	−	F	w	+	+	+	M	+
Urease		−	−	−	−	−	+	−	−	−	−
Alkaline phosphatase		M	−	−	−	−	V	U	U	−	−
Hippuricase		+	+	+	M	+	+	V	M	+	+
Indoxyl acetate hydrolysis		−	−	−	−	+	+	+	+	+	M
Reduction:											
	Nitrate	+	V	V	V	+	+	F	V	−	+
	Selenite	+	+	+	+	−	−	U	U	−	M
	TTC	M	+	+	+	−	+	U	U	V	M
H_2_S production on TSI		−	−	V	V	−	V	−	−	−	−
α-Haemolysis		−	−	−	−	−	−	−	−	+	+
Growth on:											
	2 % (w/v) NaCl	−	−	−	−	−	+	−	−	−	−
	1 % (w/v) glycine	V	−	M	F	−	+	+	+	F	M
	0.04 % (w/v) TTC	M	+	+	+	U	−	V	+	V	M
	mCCDA	w	+	+	+	−	+	U	U	+	+
	0.032 % (w/v) methyl orange	+	+†	+†	+†	U	U	U	U	+	+
	0.1 % (w/v) TMAO (anaerobic)	+	+†	+†	+†	U	U	U	U	−	−
Resistance to:											
	Nalidixic acid (30 mg l^−1^)	S	V	S	V	S	R	S	V	S	S
	Cephalothin (30 mg l^−1^)	R	R	R	R	R	R	V	R	S	V

Taxa: 1, *Campylobacter molothri* sp. nov. (*n* = 28); 2, *Campylobacter taeniopygiae*; 3, *Campylobacter aviculae*; 4, *Campylobacter estrildidarum*; 5, *Campylobacter avium*; 6, *Campylobacter canadensis*; 7, *Campylobacter bilis*; 8, *Campylobacter hepaticus*; 9, *Campylobacter jejuni* subsp. *doylei* and 10, *Campylobacter jejuni* subsp. *jejuni*; Positive strains: + (95−100%), M (70−95%), V (30−70%), F (10−30%), − (0−10%); w, weak growth; for antibiotic resistance, S/V/R indicates sensitive, variable or resistant, respectively; U, unknown/not determined. The complete comparison within the genus *Campylobacter* is shown in Table S2. All taxa could grow microaerobically at 37 °C but could not grow aerobically at 37 °C, and were oxidase positive. Data for columns 2−10 are derived from the original species descriptions, On et al., 2017 [23] or Miller et al., 2024 [19]. †: phenotypes tested in this study.

To further characterize *C. molothri*, the genomes of all 29 strains were sequenced. The strains were grown microaerobically at 37 °C for 48 h on brain–heart infusion agar (Thermo Fisher Scientific, Waltham, MA) supplemented with 5% laked horse blood. Genomic DNAs were prepared from these cultures using the Promega Wizard Genomic DNA Purification Kit. A gap-free, complete genome of RM10537^T^ was obtained using a Pacific Biosciences RSII sequencer, as described earlier [24]. Illumina HiSeq reads for strain RM10537^T^ were obtained from SeqWright (Houston, TX). These reads were added to the assembly to improve base calling. The genome sequences of the remaining 28 strains were determined to the draft level using assembled Illumina MiSeq reads, as described earlier [25]. Sequencing metrics and accession numbers for the genomes (GenBank) and MiSeq reads (NCBI Sequence Read Archive or European Nucleotide Archive) are provided in Table S3. The strain RM10537^T^ genome is 1.5134 Mbp (Table S4) and also contains a 3526 bp plasmid that is >97 % similar to the *C. jejuni* plasmid pTIW94 [26] and >95 % similar to the *C. coli* plasmid pCC2228-2 [27]; the plasmid carriage of the other 28 strains was not determined. DNA G+C contents for *C. molothri* range from 28.1 to 28.3 mol% (Table S3), which are the lowest reported within *Campylobacter*, except for *C. novaezeelandiae* (27.5 mol% [28]) and *C. hepaticus* (27.9 mol% [13]). These values are also distinct from the reported DNA G+C contents of the related taxa *C. aviculae*, *C. estrildidarum* and *C. taeniopygiae* [14] (29.6, 29.2 and 29.4 mol%, respectively). The type strain genome contains 18 GC tracts ≥8 bp; 17 of these were determined to be hypervariable using the HiSeq reads, as described earlier [29]. Annotation of the strain RM10537^T^ genome was performed, as described earlier [30]. The predicted proteomes were extracted from the 28 draft genomes, compiled and compared to the strain RM10537^T^ proteome by blastp to determine gene presence/absence throughout the strain set.

**Fig. 2.**
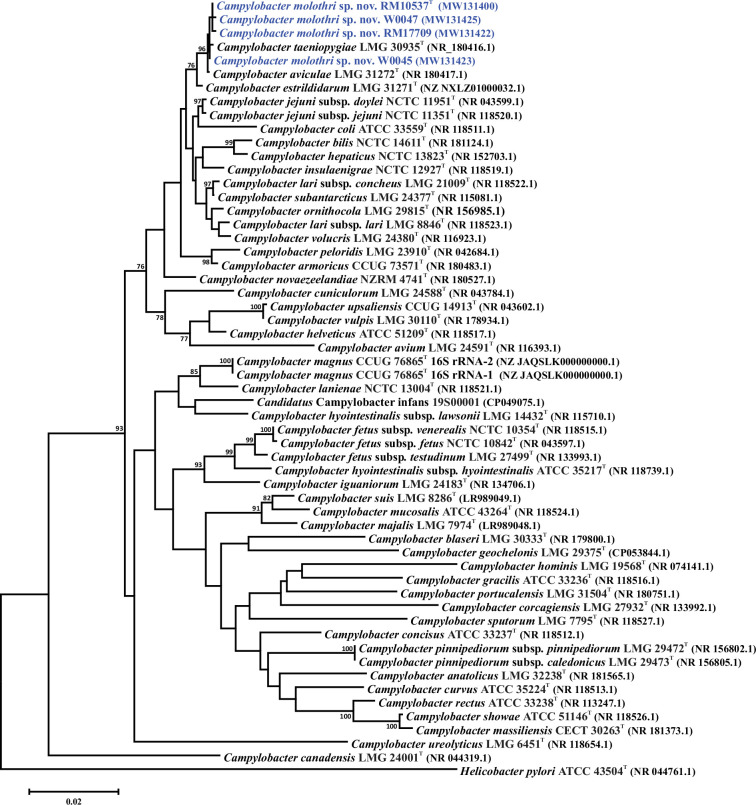
16S rRNA gene sequence phylogenetic tree representing *Campylobacter molothri* sp. nov. and the *Campylobacter* taxa type strains. 16S rRNA gene accession numbers are appended parenthetically to each node. Bootstrap values >75 % are shown next to the branches. The scale bar represents the number of base substitutions per site. The 16S rRNA gene sequence of the *Helicobacter pylori* type strain was used to root the tree. The 16S rRNA gene sequences of strains W0045, W0047 and RM17709 have 3, 1 and 1 SNPs relative to the 16S rRNA gene sequence of *Campylobacter molothri* sp. nov. RM10537^T^; the 16S rRNA gene sequences of the remaining *Campylobacter molothri* sp. nov. strains are identical to those of the type strain and are not included in the dendrogram.

**Fig. 3.**
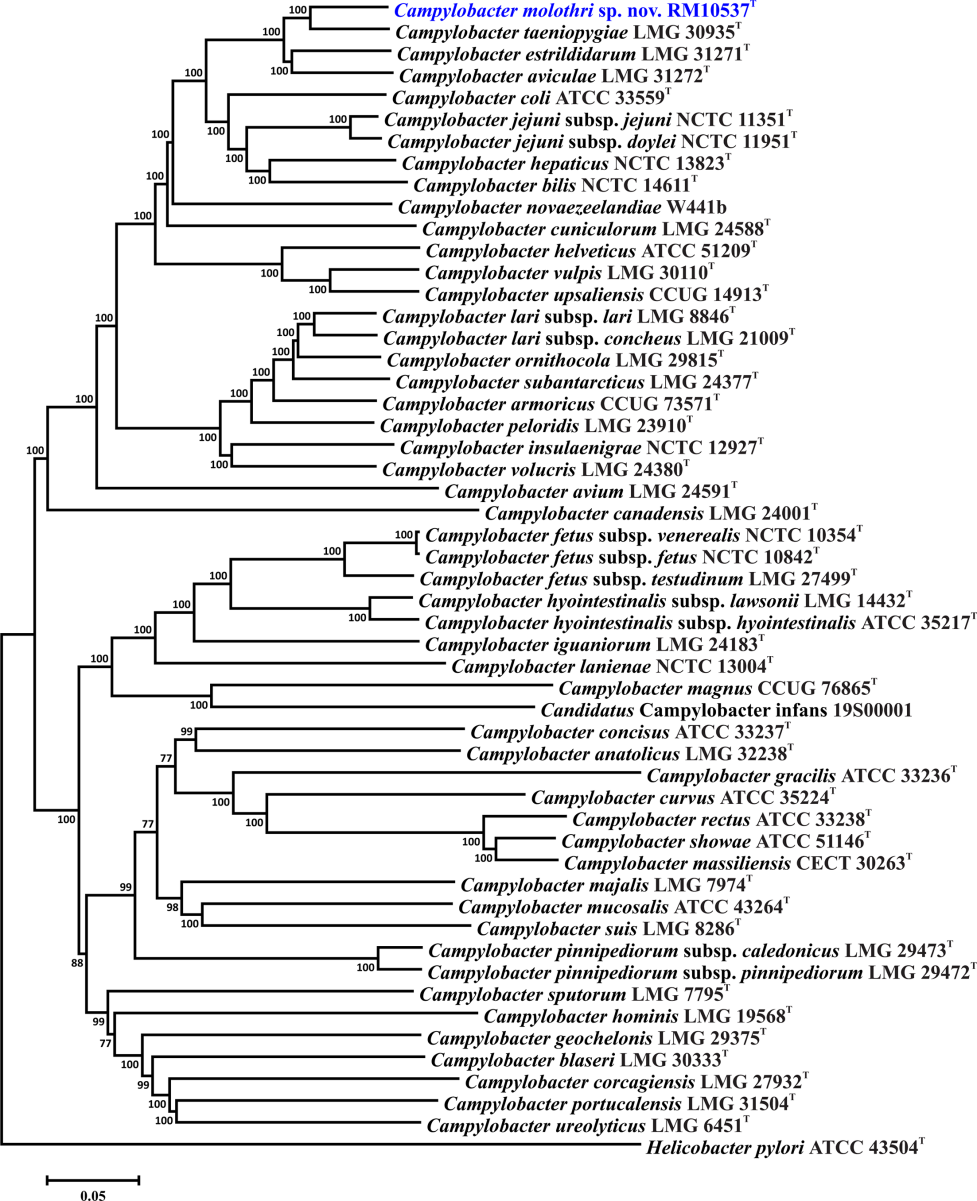
Core-genome phylogenetic tree representing *Campylobacter molothri* sp. nov. and the *Campylobacter* taxa type strains. Bootstrap values >75 % are shown at the nodes. The *Helicobacter pylori* type strain core gene sequences were concatenated and used in the alignment to root the tree. The scale bar represents the number of base substitutions per site.

The taxonomic position of the 29 *C*. *molothri* strains was determined through single-gene and whole-genome comparisons, using the 16S rRNA genes and a set of 330 core genes. The *C. molothri* 16S rRNA genes were extracted from the genomes, and the 16S rRNA gene sequences of the *Campylobacter* type strains were downloaded from GenBank. The list of 330 core genes used in this analysis is shown in Table S5. The 16S rRNA gene sequences were aligned using ClustalX. The core genes were aligned individually within Geneious ver. 2022.0.1 using Clustal Omega; the 330 alignments were then concatenated alphabetically by gene name into a single alignment. Phylogenetic trees were constructed using mega ver. 6 [31], the neighbour-joining method [32], the Kimura 2-parameter distance estimation method [33] and 1000 bootstrap replicates. Four different *C. molothri* 16S rRNA gene sequences were identified within the set of 29 strains (Fig. 2). These four 16S rRNA gene sequences could not be distinguished from the 16S rRNA gene sequences of the *C. taeniopygiae* or *C. aviculae* type strains. This is consistent with other studies, where it was shown that different, but related, *Campylobacter* species can have a very high degree of similarity at the 16S rRNA locus [14,34, 35]. However, whole-genome comparisons using the core gene set demonstrated that *C. molothri* RM10537^T^ was well separated from the type strains of the related zebra finch-associated species (Fig. 3). Using the genes of the PubMLST *C. lari* typing scheme [36], 27 different *C. molothri* sequence types were identified, indicating a substantial amount of diversity within the strain set (Table S1). Nevertheless, expanding the analysis to include the core genomes of the other 28 *C*. *molothri* strains did not change the above results: all 29 *C*. *molothri* strains could be distinguished from the related *Campylobacter* taxa (Fig. S1).

Pairwise whole-genome comparisons were performed between the *C. molothri* type strain and the genomes of the *Campylobacter* type strains using average nucleotide identity (ANI) and digital DNA–DNA hybridization (dDDH) analyses. DNA–DNA hybridization has been used historically for species delineation, but DDH has been largely supplanted by more *in silico* methods, such as ANI analysis and dDDH, where the DDH species boundary value of 70% is approximately equivalent to an ANI value of 95% [37,38]. ANI analyses were performed using JSpecies (ver. 1.2.1; [38]). All pairwise comparisons between *C. molothri* strain RM10537^T^ and related *Campylobacter* taxa were below the 95% species boundary (Table 2). *Campylobacter taeniopygiae* was the most closely related species, with an ANI value of 90.9%; no other *Campylobacter* species had a pairwise ANI value >87 %. ANI analysis using all 29 *C. molothri* strains and all *Campylobacter* type strains yielded similar results (Table S6). dDDH analyses were performed using the Genome-to-Genome Distance Calculator (GGDC ver. 2.1 https://ggdc.dsmz.de/ggdc.php# [39]). Pairwise dDDH values between the *C. molothri* type strain and related *Campylobacter* taxa were <67% using GGDC formula 3 (Table 2) and were therefore consistent with the ANI analysis. Together, the ANI and dDDH analyses indicate that *C. molothri* represents a novel species within *Campylobacter* since the values from both analyses are below the species cut-off values.

**Table 2.** Pairwise ANI and dDDH values between *Campylobacter molothri* sp. nov. and related *Campylobacter* taxa. Pairwise ANI values between the *Campylobacter molothri* type strain and other *Campylobacter* taxa >74% and pairwise dDDH values between the *Campylobacter molothri* type strain and other *Campylobacter* taxa >18% are shown. The full pairwise ANI comparison between *Campylobacter molothri* sp. nov. and other *Campylobacter* taxa is shown in Table S6

ANI	1	2	3	4	5	6	7	8	9	10	11
1, *C. molothri*	–	90.9	86.5	86.3	79.4	79.4	79.3	79.1	78.2	76.6	74.9
2, *C. taeniopygiae*	90.8	–	88.3	87.3	79.5	79.3	79.4	78.6	78.1	76.0	75.1
3, *C. aviculae*	86.3	88.2	–	88.5	79.7	79.6	79.8	78.9	78.3	76.0	74.6
4, *C. estrildidarum*	86.2	87.4	88.6	–	79.6	79.7	79.7	79.0	78.4	76.2	74.9
5, *C. jejuni* subsp*. jejuni*	79.3	79.6	79.8	79.8	–	96.1	84.1	84.0	83.8	76.7	76.2
6, *C. jejuni* subsp. *doylei*	79.2	79.4	79.7	79.9	96.1	–	84.0	83.6	83.7	77.0	75.6
7, *C. coli*	79.2	79.4	79.8	79.7	84.0	83.9	–	80.4	80.2	76.3	75.4
8, *C. hepaticus*	79.0	78.7	79.2	79.2	84.1	83.8	80.6	–	85.1	76.7	75.3
9, *C. bilis*	78.1	78.1	78.4	78.4	83.9	83.7	80.2	85.1	–	75.7	74.7
10, *C. novaezeelandiae*	76.4	75.9	76.0	76.1	76.7	76.8	76.2	76.4	75.8	–	74.9
11, *C. cuniculorum*	74.9	75.3	75.1	75.2	76.3	75.7	75.6	75.3	74.8	75.0	–
	**RM10537^T^**										
**dDDH**	**dDDH**		**Model3 C.I.**								
*C. taeniopygiae*	66.4		[63.0–69.7%]								
*C. estrildidarum*	59.4		[56.2–62.6%]								
*C. aviculae*	56.3		[53.1–59.4%]								
*C. jejuni* subsp. *jejuni*	34.7		[31.7–37.8%]								
*C. hepaticus*	33.6		[30.6–36.6%]								
*C. coli*	31.4		[28.5–34.5%]								
*C. jejuni* subsp. *doylei*	30.6		[27.7–33.7%]								
*C. bilis*	26.5		[23.6–29.6%]								
*C. novaezeelandiae*	24.0		[21.2–27.1%]								

Analysis of the *C. molothri* genomes indicated that multiple genes associated with amino acid/cofactor biosynthesis and the TCA cycle were consistently absent from all 29 strains, a feature previously described for members of the *Campylobacter lari* group [29]. Within the *C. lari* group, genes involved in the biosynthesis of arginine, leucine, methionine, pantothenate, proline and tryptophan are absent, thus making these strains likely auxotrophs for these amino acids and vitamins ([29], see also Table S7). In *C. molothri*, these genes are also absent; however, genes involved in cysteine (*cysEK*), histidine (*hisA*(*BJ*)*CDF*(*G_L_*)*H*(*IE*)), isoleucine/valine (*ilvACDEHI*), phenylalanine (*pheA*), serine (*serA*) and tyrosine (*tyrA*) biosynthesis were also not identified in any of the *C. molothri* genomes (Table S7). The predicted absence of so many amino acid and cofactor biosynthetic pathways is striking, suggesting that *C. molothri* synthesizes very few amino acids *de novo*. The only multi-gene amino acid biosynthetic pathway encoded by *C. molothri* is the lysine biosynthetic pathway (Table S7). Thus, the only amino acids synthesized *de novo* by *C. molothri* may be aspartate, glutamate, glutamine, glycine and lysine. Interestingly, the amino-acid biosynthetic genes absent in *C. molothri* are also largely absent from the type strain genomes of the related zebra finch-associated taxa (Table S7). Although alternate biosynthetic pathways may exist in these species, it is likely that they acquire many amino acids from either the breakdown of peptides or proteins or via the diet or intestinal microbiota of their avian hosts. TCA cycle genes absent in *C. molothri* (i.e., *acnB*, *gltA*, *icd* and *sucCD*) were also not identified in the *C. lari* group genomes [29]. As with the amino acid biosynthetic pathways, these TCA cycle genes were also not identified within *C. taeniopygiae* strain LMG 30935^T^ (Table S7); however, AcnB, GltA and Icd are encoded by the related *C. aviculae* and *C. estrildidarum* type strains. Additionally, although *C. molothri* sp. nov. encodes the membrane-associated Mqo malate:quinone-oxidoreductase, it does not encode the NAD-dependent Mdh malate dehydrogenase, a feature also observed within *C. insulaenigrae* of the *C. lari* group.

Genes encoding components of the Entner–Doudoroff (E-D) pathway (termed the *glc* locus by Vorwerk *et al*., [40]) were also identified in *C. molothri* sp. nov. (Table S7). *Campylobacter* are noteworthy for their inability to catabolize glucose, which is primarily due to two defects in the Embden–Meyerhoff–Parnas glycolytic pathway, specifically the lack of glucokinase and phosphofructokinase. However, the E-D pathway represents an alternative to glycolysis, bypassing the blockage at the phosphofructokinase step. Indeed, *Campylobacter* strains possessing the *glc* locus have been shown to catabolize glucose [40,41]. The *glc* locus is rare in *Campylobacter* [41] but has been identified in some *C. jejuni* [41,42], *C. coli* [40,41] and * C. cuniculorum* [43] strains and also in the genomes of *C. hepaticus*, *C. bilis* and *C. canadensis* (data not shown). In *C. jejuni*, * C. coli* and *C. hepaticus*, the *glc* locus is colocalized to a ribosomal RNA operon, in which the E-D genes are present between the tRNA^Ile^ and 23S rRNA genes. These ribosomal loci are linked to either *ansB* (e.g. *C. jejuni* subsp. *doylei* strain NCTC 11951^T^) or *cjaC* (e.g., *C. jejuni* subsp. *doylei* strain 269.97). The *C. molothri* sp. nov. strain RM10537^T^ *glc* locus is within the second *cjaC*-linked ribosomal operon; the chromosomal position of the *glc* loci in the other 28 strains could not be determined. A unique feature identified in the *C. molothri* type strain genome is the presence of additional genes associated with galactose metabolism (Table S7). These genes, *galK, galM* and *galT*, encode components of the Leloir pathway. This pathway converts β-d-galactose into glucose-6-phosphate [44], which can then be further catabolized via the E-D pathway. *galE,* which encodes a UDP-glucose 4-epimerase, was not identified; however, *C. molothri* does encode Gne, which has been shown to have a dual UDP-glucose 4-epimerase/UDP-*N*-acetylglucosamine 4-epimerase function in *C. jejuni* [45]. Two of the Leloir pathway genes, *galK* and *galT*, are directly adjacent to *eda* in the *glc* locus. The third gene, *galM,* is linked to a type II-C CRISPR/Cas locus. Notably, the *glc* locus and Leloir pathway genes were also identified here in the type strain genomes of the Zebra finch-associated species (Table S7). Therefore, *C. molothri* is one of four highly related hippuricase-positive and potentially glucose/galactose-utilizing species that form a discrete clade within *Campylobacter*. Strains from these species were recovered from passerine birds with a common diet of seeds and insects/invertebrates. Further research will be needed to determine if their unique gene content plays a role in the colonization of their passerine hosts.

The lipooligosaccharide (LOS) locus of the *C. molothri* type strain genome contains four genes reported previously to be involved in the biosynthesis (*neuBCA*) and transfer (*cst*) of sialic acid in *C. jejuni* [46,48]. In *C. jejuni*, the production of sialylated LOS in some strains may lead to the autoimmune paralytic disorder Guillain–Barré syndrome (GBS) following infection [49,50]. The *cst* gene of strain RM10537^T^ contains a hypervariable GC tract at its 5’ end and is likely a contingency gene. Eleven other *C. molothri* strains (RM9754, RM9759, RM9760, RM9929, RM9930, RM10532, RM10534, RM10538, RM10542, W0046 and W0047) also possess similar *cst-neuBCA* clusters, and GC tracts were identified in these *cst* genes. A *neuBCA* cluster was identified here in the *C. taeniopygiae* type strain genome; however, the cognate sialyltransferase (*cst*) was not present in the draft genome. The *cst^+^ C. molothri* strains were recovered from cowbirds, blackbirds and reed buntings in California and Scotland, indicating that carriage of *cst-neuBCA* is not restricted by host or geography. Although the requisite genes are present, additional experiments would need to be performed to determine whether these strains produce sialylated LOS that mimic human gangliosides. Additionally, although it is unlikely that *C. molothri* directly leads to human illness, given its current host population, passerine birds are commonly found in or around livestock and poultry production facilities. Thus, additional work is certainly warranted on the epidemiology of *C. molothri* and its potential as a causative agent of human illness and GBS.

## Description of *Campylobacter molothri* sp. nov.

*Campylobacter molothri* (mo.lo’thri. N.L. gen. n. *molothri*, of *Molothrus ater*, the source of the type strain). Cells are Gram-negative, curved or spiral rods, with an average width of 0.22 µm and an average length of 2.12 µm. Cells are motile with bipolar flagella. Some coccoid cells are observed. Growth occurs on ABA-B at both 37 and 42 °C under microaerobic conditions and at 37 °C under anaerobic conditions. No growth on ABA-B at 30 °C under microaerobic conditions or at any temperature under aerobic conditions. All strains demonstrate oxidase, catalase and hippuricase activity but no urease activity. Most strains (22/28; 79%) demonstrate alkaline phosphatase activity. Does not hydrolyse indoxyl acetate. All strains reduce nitrate but not selenite; most strains (25/28; 89%) reduce TTC. Growth is not supported on ABA-B with a final NaCl concentration ≥2% (w/v). Growth on ABA-B amended with 1% (w/v) glycine is variable (14/28; 50%). Most strains grow on ABA-B amended with 0.04% TTC (25/28; 89%). All strains grow on media amended with 0.032% (w/v) methyl orange and anaerobically on media amended with 0.1% (w/v) TMAO. Strains are resistant to 30 mg l^−1^ cephalothin but sensitive to 30 mg l^−1^ nalidixic acid. The type strain is RM10537^T^ (=LMG 32306^T^=CCUG 75331^T^), which was recovered from a brown-headed cowbird in 2009. *Campylobacter molothri* sp. nov. strain RM12397 has also been deposited into the LMG culture collection (=LMG 32307). Pathogenicity is unknown. Accession numbers for the 16S rRNA gene and genome sequence of the type strain are MW131400 and CP059597, respectively.

## supplementary material

10.1099/ijsem.0.006635 Uncited Supplementary Material 1.

10.1099/ijsem.0.006635 Uncited Supplementary Material 2.

10.1099/ijsem.0.006635 Uncited Supplementary Material 3.

## Abbreviations

ABA: anaerobe basal agar
ANI: average nucleotide identity
CAT: cefoperazone–amphotericin–teicoplanin
dDDH: digital DNA-DNA hybridization
E-D: Entner–Doudoroff
GGDC: Genome-to-Genome Distance Calculator
LOS: lipooligosaccharide
mCCDA: modified charcoal–cefoperazone–deoxycholate agar
MLST: multilocus sequence typing
TMAO: trimethylamine *N*-oxide
TSI: triple sugar iron
TTC: triphenyltetrazolium chloride
