# Supplementary material for: Campylobacter molothri sp. nov. isolated from wild birds
Source: Int J Syst Evol Microbiol. 2025 Feb 6;75(2):006635. doi: 10.1099/ijsem.0.006635 (PMC11801493; doi:10.1099/ijsem.0.006635)
Supplement: Uncited Supplementary Material 1. [file ijsem-75-06635-s001.pdf]

Figure S1: Core gene phylogenetic tree representing the 29 *Campylobacter molothri* and related *Campylobacter* type strains. Dendrogram construction is the same as described in Figure 2. Bootstrap values >75%, generated from 1000 replicates, are shown at the nodes. The *Campylobacter upsaliensis* type strain core gene sequences were concatenated and used in the alignment to root the tree. The scale bar represents the number of base substitutions per site.

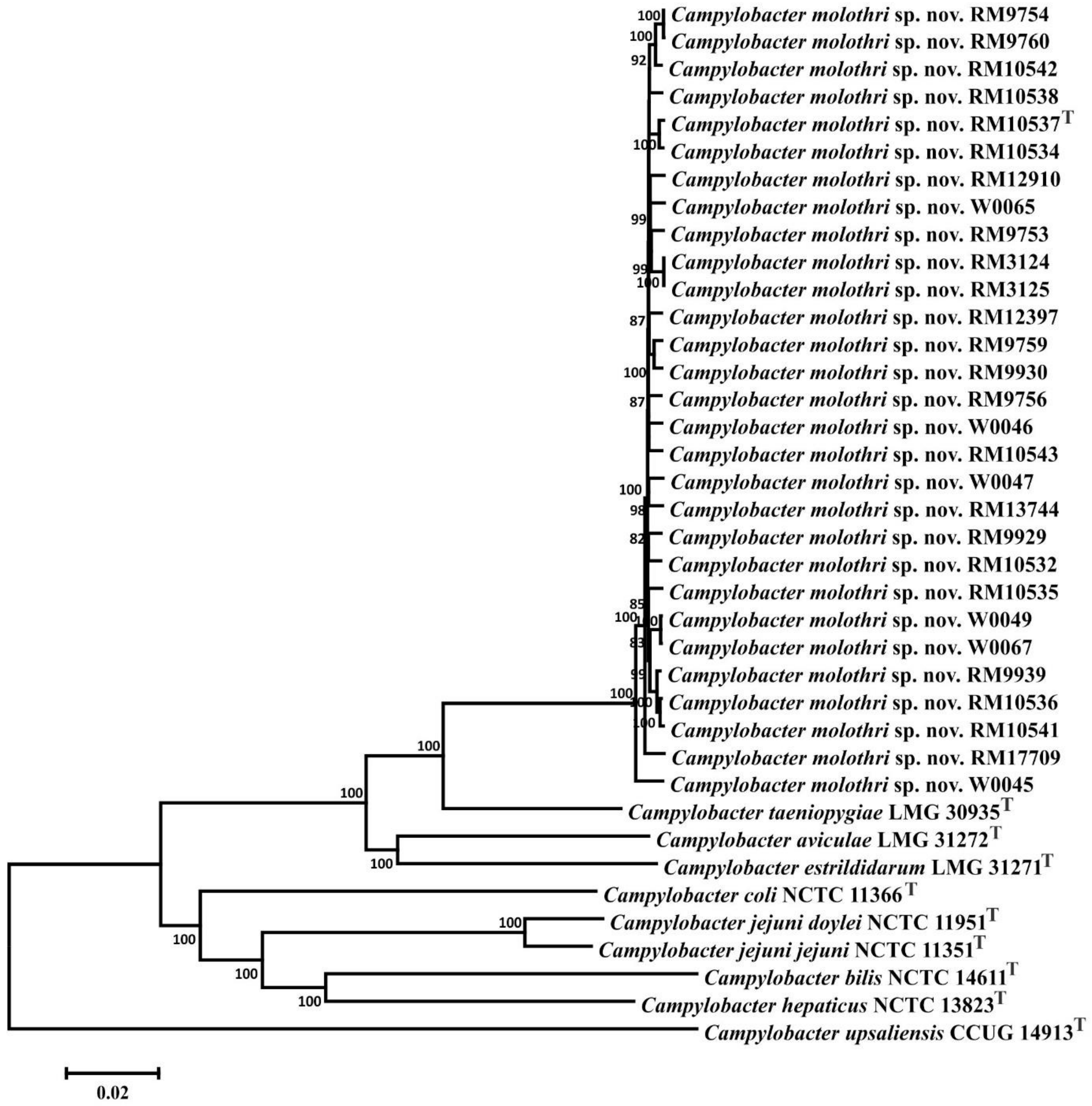

Table S1: Strain data for *C. molothri* sp. nov. Multi-locus sequence typing used the seven genes from the *C. lari* MLST scheme, i.e., *adk, atpA, glnA, glyA, pgi, pgm* and *tkt.* MLST genes were extracted from the complete or draft genomes and allele endpoints were identified following alignment with *C. lari* reference alleles. Since *C. molothri* strains do not contain *gltA*, the *C. lari* MLST scheme was used in place of the *C. jejuni/C. coli* scheme. Sequence types are arbitrary for this study and were not deposited into PubMLST.

**Strain**	**Source**	**Date**	**Isolation locale**	**MLST ST**
RM10537^{T}	Brown-headed cowbird (*Molothrus ater*)	13 Nov 2009	USA: California: Monterey County	ST-1
RM3124	Creek water	19 Apr 2002	USA: California: Sonoma Creek	ST-15
RM3125	Creek water	19 Apr 2002	USA: California: Sonoma Creek	ST-15
RM9753	Black bird (*Euphagus cyanocephalus*)	01 Sep 2009	USA: California: Monterey County	ST-16
RM9754	Black bird (*Euphagus cyanocephalus*)	01 Sep 2009	USA: California: Monterey County	ST-7
RM9756	Black bird (*Euphagus cyanocephalus*)	01 Sep 2009	USA: California: Monterey County	ST-13
RM9759	Black bird (*Euphagus cyanocephalus*)	01 Sep 2009	USA: California: Monterey County	ST-5
RM9760	Black bird (*Euphagus cyanocephalus*)	01 Sep 2009	USA: California: Monterey County	ST-8
RM9929	Black bird (*Euphagus cyanocephalus*)	15 Sep 2009	USA: California: Monterey County	ST-4
RM9930	Black bird (*Euphagus cyanocephalus*)	15 Sep 2009	USA: California: Monterey County	ST-10
RM9939	Black bird (*Euphagus cyanocephalus*)	15 Sep 2009	USA: California: Monterey County	ST-21
RM10532	Brown-headed cowbird (*Molothrus ater*)	13 Nov 2009	USA: California: San Benito County	ST-12
RM10534	Brown-headed cowbird (*Molothrus ater*)	13 Nov 2009	USA: California: Monterey County	ST-2
RM10535	Brown-headed cowbird (*Molothrus ater*)	13 Nov 2009	USA: California: Monterey County	ST-25
RM10536	Brown-headed cowbird (*Molothrus ater*)	13 Nov 2009	USA: California: Monterey County	ST-20
RM10538	Black bird (*Euphagus cyanocephalus*)	13 Nov 2009	USA: California: Monterey County	ST-24
RM10541	Brown-headed cowbird (*Molothrus ater*)	18 Nov 2009	USA: California: Monterey County	ST-19
RM10542	Black bird (*Euphagus cyanocephalus*)	18 Nov 2009	USA: California	ST-6
RM10543	Black bird (*Euphagus cyanocephalus*)	18 Nov 2009	USA: California: Monterey County	ST-9
RM12397	Wild bird	27 May 2010	USA: California: San Luis Obispo County	ST-14
RM12910	Wild bird	30 Jul 2010	USA: California: San Benito County	ST-18
RM13744	Wild bird	19 Nov 2010	USA: California: San Benito County	ST-23
RM17709	Environmental water	07 Apr 2014	USA: California: Salinas River/King City	ST-27
W0045	Dunnock (*Prunella modularis*)	10 Mar 2015	UK: Scotland: Angus: Crombie Country Park	ST-26
W0046	Reed bunting (*Emberiza schoeniclus*)	19 Mar 2015	UK: Scotland: Angus: Montrose Basin: The Lurgies	ST-3
W0047	Reed bunting (*Emberiza schoeniclus*)	19 Mar 2015	UK: Scotland: Angus: Montrose Basin: The Lurgies	ST-11
W0049	Reed bunting (*Emberiza schoeniclus*)	19 Mar 2015	UK: Scotland: Angus: Montrose Basin: The Lurgies	ST-22
W0065	Yellowhammer (*Emberiza citronella*)	23 Mar 2015	UK: Scotland: Angus: Montrose Basin: The Lurgies	ST-17
W0067	Reed bunting (*Emberiza schoeniclus*)	23 Mar 2015	UK: Scotland: Angus: Montrose Basin: The Lurgies	ST-22

Table S2. Phenotypic characteristics of *C. molothri* sp. nov. and other *Campylobacter* taxa.

	molothri sp. nov.	*anatolicus*	*armoricus*	*aviculae*	*avium*	*bilis*	*blaseri*	*canadensis*	*coli*	*concisus*	*corcagiensis*	*cuniculorum*	*curvus*	*devanensis*	*estrildidarum*	*fetus fetus*	*fetus venerealis*	*fetus testudinum*	*geochelonis*	*gracilis*	*helveticus*	*hepaticus*	*hominis*	*hyo. hyointestinalis*	*hyo. lawsonii*	*iguaniorum*	*insulaenigrae*	*jejuni doylei*
Motility	+	+	+	+	+	+	−	+	+	+	−	+	+	+	+	+	+	+	−	−	+	+	−	+	+	+	+	+
Temperature (atmosphere)																												
37 °C (aerobic)	−	−	−	−	−	−	−	−	−	−	−	−	−	−	−	−	−	F	−	−	−	−	−	−	−	−	−	−
30 °C (microaerobic)	−	+	−	−	−	U	+	−	−	M	+	U	+	−	−	+	+	+	+	M	V	U	−	+	+	+	−	−
37 °C (microaerobic)	+	+	+	+	+	+	+	+	+	+	+	+	+	+	+	+	+	+	+	+	+	+	+	+	+	+	+	+
42 °C (microaerobic)	+	+	+	+	+	+	+	+	+	M	+	M	M	+	+	M	−	M	−	M	+	+	−	+	+	−	−	−
37 °C (anaerobic)	+	+	+	+	−	−	+	+	−	+	+	−	+	+	+	V	M	+	+	+	−	−	+	−	+	w	−	−
Oxidase	+	+	+	+	+	+	+	+	+	V	+	+	+	+	+	+	+	+	+	−	+	+	+	+	+	+	+	+
Catalase	+	−	+	−	w	+	+	+	+	−	+	+	−	+	F	+	+	+	+	V	−	+	−	+	+	+	+	M
Urease	−	−	+	−	−	−	+	+	−	−	+	−	−	−	−	−	−	−	−	−	−	−	−	−	−	−	−	−
Alkaline phosphatase	M	+	−	−	−	U	+	V	−	M	+	−	V	+	−	−	−	−	−	−	−	U	−	−	F	+	U	−
Hippuricase	+	−	−	+	+	V	−	+	−	−	−	−	−	−	M	−	−	−	+	−	−	M	−	−	−	−	−	+
Indoxyl acetate hydrolysis	−	−	−	−	+	+	+	+	+	−	V	+	V	+	−	−	−	−	−	M	+	+	−	−	−	−	−	+
Reduction:																												
Nitrate	+	−	−	V	+	F	+	+	+	F	M	+	+	+	V	+	M	+	+	M	+	V	V	+	+	+	+	−
Selenite	+	U	V	+	−	U	U	−	+	F	U	−	−	+	+	M	F	+	−	−	−	U	−	+	+	−	+	−
TTC	M	−	V	+	−	U	U	+	+	−	U	V	V	+	+	−	−	+	−	−	−	U	−	F	−	+	+	V
H_2S production on TSI	−	+	U	V	−	−	+	V	−	−	+	−	F	−	V	−	−	−	−	−	−	−	−	+	+	+	−	−
α−haemolysis	−	−	−	−	−	−	−	−	F	F	−	+	F	−	−	−	V	−	−	−	+	−	−	V	V	+	U	+
Growth on:																												
2% (w/v) NaCl	−	+	−	−	−	−	U	+	−	F	+	−	V	+	−	−	−	−	+	V	F	−	U	−	−	−	−	−
1% (w/v) glycine	V	+	+	M	−	+	w	+	M	F	+	−	+	+	F	+	F	+	+	+	V	+	+	+	F	+	+	F
0.04% (w/v) TTC	M	U	V	+	U	V	U	−	+	−	−	V	+	−	+	−	−	+	U	−	−	+	−	F	−	−	+	V
mCCDA	+	+	U	+	−	U	−	+	+	F	U	M	M	+	+	+	+	+	+	V	+	U	U	+	+	+	U	+
0.032% (w/v) methyl orange	+	U	U	+†	U	U	U	U	U	M	U	U	V	U	+†	+	M	U	U	V	V	U	U	+	+	U	−	+
0.1% (w/v) TMAO	+	U	U	+†	U	U	U	U	U	M	U	U	+	U	+†	F	F	U	U	+	−	U	U	M	M	U	V	−
Resistance to:																												
Nalidixic acid (30 mg L^{-1})	S	R	S	S	S	S	S	R	S	V	R	V	R	R	V	R	V	R	R	V	S	V	V	R	R	R	R	S
Cephalothin (30 mg L^{-1})	R	R	R	R	R	V	S	R	R	S	S	V	S	R	R	S	S	R	S	S	S	R	S	V	S	S	R	S

Positive: + (95-100%); M (70–95%); V (30–70%); F (10–30%); − (0-10%); w: weak growth/reaction; U: unknown/not determined

Resistance: S, R and V indicate sensitive, resistant and variable, respectively. §, urease-positive thermophilic campylobacters (UPTC)

Data are derived from the original species descriptions and/or Boukerb et al., *Int J Syst Evol Microbiol* 2019;69:3969-3979, On et al., *Int J Syst Evol Microbiol* 2017;67:5296-5311, Parisi et al., *Syst Appl Microbiol* 2021;44:126204 or Miller et al., *Int J Syst Evol Microbiol* 2024;74:006405

†: phenotype determined in this study

	jejuni jejuni	*lanienae*	*lari concheus*	*lari lari*	*magnus*	*majalis*	*massiliensis*	*mucosalis*	*novaezeelandiae*	*ornithocola*	*peloridis*	*pin. caledonicus*	*pin. pinnipediorum*	*porcelli*	*portucalensis*	*rectus*	*showae*	*sput. fecalis*	*sput. paraureolyticus*	*sput. sputorum*	*subantarcticus*	*suis*	*taeniopygiae*	*upsaliensis*	*ureolyticus*	*vicugnae*	*volucris*	*vulpis*
Motility	+	+	+	+	+	U	−	+	+	+	+	+	+	+	−	+	+	+	+	+	+	U	+	+	−	+	+	+
Temperature (atmosphere)																												
37 °C (aerobic)	−	−	−	−	−	−	−	−	−	−	−	−	−	−	−	−	−	−	−	−	−	w	−	−	−	−	−	−
30 °C (microaerobic)	M	−	−	+	−	−	U	+	U	−	−	+	+	−	U	F	+	M	M	M	−	−	−	+	+	−	−	−
37 °C (microaerobic)	+	+	+	+	+	+	+	+	+	+	+	+	+	+	+	+	+	+	+	+	+	+	+	+	+	+	+	+
42 °C (microaerobic)	+	+	+	+	+	+	−	+	+	+	+	−	−	+	+	F	V	V	V	V	+	+	+	+	V	+	+	+
37 °C (anaerobic)	−	w	−	−	+	+	+	+	+	+	−	+	+	+	w	+	+	+	+	+	+	+	+	−	+	+	+	−
Oxidase	+	+	+	+	+	−	U	+	+	+	+	+	+	+	+	+	V	+	+	+	+	−	+	+	+	+	+	+
Catalase	+	+	+	+	+	−	U	−	+	+	+	−	+	+	−	F	V	+	−	−	+	−	+	−	F	+	+	−
Urease	−	−	−	V§	−	−	−	−	−	+	−	+	+	−	−	−	−	−	+	−	−	−	−	−	+	−	−	−
Alkaline phosphatase	−	+	U	−	V	U	−	M	−	−	−	U	U	+	U	−	−	−	−	−	U	U	−	−	−	+	−	V
Hippuricase	+	−	−	−	−	−	−	−	−	−	−	−	−	−	−	−	−	−	−	−	−	−	+	−	−	−	−	−
Indoxyl acetate hydrolysis	M	−	U	F	+	−	U	−	+	−	−	−	−	−	−	+	V	−	−	−	−	−	−	+	F	−	−	+
Reduction:																												
Nitrate	+	+	+	+	+	−	−	F	+	V	+	+	+	+	−	+	+	M	+	+	+	−	V	+	+	+	+	+
Selenite	M	V	U	V	+	U	U	F	−	U	U	U	U	+	U	−	−	V	V	V	−	U	+	+	−	−	+	+
TTC	M	+	U	M	−	U	−	−	F	F	−	U	U	+	U	−	−	−	−	−	U	U	+	V	−	+	−	−
H_2S production on TSI	−	−	U	−	−	+	−	+	−	−	U	+	+	−	−	−	V	+	+	+	−	−	−	−	−	−	−	−
α−haemolysis	+	+	U	+	−	U	U	−	+	−	U	+	+	−	−	+	+	+	+	+	+	U	−	+	V	−	U	+
Growth on:																												
2% (w/v) NaCl	−	−	+	M	−	−	U	M	+	U	M	U	U	+	−	V	+	+	+	+	+	−	−	−	+	−	−	−
1% (w/v) glycine	M	−	+	+	−	−	U	V	+	+	+	−	V	+	V	+	V	+	+	+	M	−	−	+	+	−	−	+
0.04% (w/v) TTC	M	V	U	M	+	−	U	−	F	U	U	U	U	−	−	−	−	−	−	−	U	−	+	V	−	−	−	U
mCCDA	+	+	+	+	+	+	U	+	U	U	+	−	−	+	U	−	+	M	M	M	U	+	+	+	V	+	U	+
0.032% (w/v) methyl orange	+	U	U	+	U	U	U	+	U	U	U	U	U	U	U	−	−	+	+	+	U	U	+†	+	+	U	+	U
0.1% (w/v) TMAO	−	U	U	+	U	U	U	+	U	U	U	U	U	U	U	+	V	M	M	M	+	U	+†	−	+	U	U	U
Resistance to:																												
Nalidixic acid (30 mg L^{-1})	S	R	S	V	R	U	S	V	S	U	V	S	S	R	U	V	S	V	V	V	R	U	V	S	S	R	R	S
Cephalothin (30 mg L^{-1})	V	R	R	R	R	U	U	V	R	U	V	S	S	R	U	S	S	S	S	S	S	U	R	V	S	V	R	S

Positive: + (95-100%); M (70–95%); V (30–70%); F (10–30%); − (0-10%); w: weak growth/reaction; U: unknown/not determined

Resistance: S, R and V indicate sensitive, resistant and variable, respectively. §, urease-positive thermophilic campylobacters (UPTC)

Data are derived from the original species descriptions and/or Boukerb et al., *Int J Syst Evol Microbiol* 2019;69:3969-3979, On et al., *Int J Syst Evol Microbiol* 2017;67:5296-5311, Parisi et al., *Syst Appl Microbiol* 2021;44:126204 or Miller et al., *Int J Syst Evol Microbiol* 2024;74:006405

†: phenotype determined in this study

Table S3: Sequencing data for the *C. molothri* sp. nov. genomes

Strain	Accession # (genome)	Accession # (reads)	Contigs (≥ 5 kbp)	Largest contig	N50	Genome size (Mb)	% G+C	Reads	Bases	Coverage (×)
RM10537^{T}	CP059597	N/A	1	N/A	N/A	1.513	28.3	18,959,115	2,422,611,208	1601
RM3124	JACHUT000000000	SRR20656718	49 (21)	161,420	123,845	1.455	28.3	1,134,940	276,581,417	190
RM3125	JACHUS000000000	SRR20656717	50 (22)	186,125	119,592	1.455	28.3	1,239,250	304,508,485	209
RM9753	JACHUR000000000	SRR20656706	48 (24)	237,758	114,738	1.461	28.2	1,274,370	311,832,890	213
RM9754	JACHUQ000000000	SRR20656697	65 (25)	232,322	111,529	1.495	28.2	1,251,780	305,223,788	204
RM9756	JACHUP000000000	SRR20656696	40 (21)	263,134	129,864	1.443	28.3	1,442,300	353,008,076	245
RM9759	JACHUO000000000	SRR20656695	51 (24)	230,547	119,152	1.471	28.2	1,209,734	296,881,159	202
RM9760	JACHUN000000000	SRR20656694	61 (23)	254,750	120,860	1.455	28.3	1,188,332	291,460,780	200
RM9929	JACHUM000000000	SRR20656692	68 (23)	224,212	155,211	1.458	28.2	1,278,254	312,144,024	214
RM9930	JACHUL000000000	SRR20656692	48 (27)	180,799	81,366	1.456	28.3	1,470,840	361,626,080	248
RM9939	JACHUK000000000	SRR20656691	56 (27)	226,605	115,687	1.497	28.2	1,814,742	445,336,382	297
RM10532	JACHUJ000000000	SRR20656716	37 (21)	219,983	112,159	1.471	28.3	1,173,438	285,549,418	194
RM10534	JACHUI000000000	SRR20656715	46 (24)	195,368	97,505	1.477	28.2	1,232,536	302,740,598	205
RM10535	JACHUH000000000	SRR20656714	50 (26)	231,092	121,743	1.461	28.2	1,369,532	335,055,216	229
RM10536	JACHUG000000000	SRR20656713	63 (33)	182,548	76,971	1.470	28.2	1,483,090	360,072,790	245
RM10538	JACHUF000000000	SRR20656712	56 (28)	173,273	83,383	1.459	28.2	1,239,722	303,970,934	208
RM10541	JACHUE000000000	SRR20656711	52 (27)	325,852	114,363	1.470	28.2	1,194,260	290,853,167	198
RM10542	JACHUD000000000	SRR20656710	57 (27)	150,939	83,453	1.456	28.3	1,233,980	302,456,598	208
RM10543	JACHUC000000000	SRR20656709	59 (25)	181,370	122,030	1.473	28.2	1,115,680	272,578,472	185
RM12397	JACHUB000000000	SRR20656708	45 (20)	290,799	138,575	1.462	28.3	1,298,408	317,979,984	217
RM12910	JACHUA000000000	SRR20656707	43 (24)	154,066	83,437	1.448	28.3	1,241,336	303,848,443	210
RM13744	JACHTZ000000000	SRR20656705	63 (24)	211,795	83,461	1.453	28.2	1,047,170	256,254,267	176
RM17709	JACHTY000000000	SRR20656705	49 (22)	247,768	107,393	1.445	28.2	1,250,960	304,266,747	211
W0045	JACHTX000000000	ERR976084	84 (29)	247,372	83,390	1.598	28.2	770,134	189,171,770	118
W0046	JACHTW000000000	ERR976085	59 (29)	231,030	83,372	1.460	28.2	1,416,496	344,895,198	236
W0047	JACHTV000000000	ERR976086	61 (25)	149,289	92,300	1.456	28.3	1,596,118	385,319,461	265
W0049	JACHTU000000000	ERR976087	60 (24)	249,625	106,340	1.486	28.1	758,636	184,727,546	124
W0065	JACHTT000000000	ERR976088	54 (22)	245,449	149,283	1.449	28.2	749,496	178,574,032	123
W0067	JACHTS000000000	ERR976246	61 (26)	185,209	83,450	1.452	28.3	1,535,826	372,026,782	256

Table S4: Genomic data for *C. molothri* strain RM10537[T]

Feature	Value(s)[a]
Genomic data	
Chromosome	
Size (Mb)[b]	1.5134
G+C content (%)	28.28
No. of CDS[c]	1,452
Defined genes (% CDS)	883 (60.8)
Specific function (% CDS)	306 (21.1)
General function/motif only (% CDS)	85 (12.3)
Hypothetical (% CDS)	178 (12.3)
Pseudogenes	30
GC tracts ≥ 8 bp (# hypervariable)	18 (17)
Plasmids	pMOL (3,526 bp)
Genomic islands/CRISPR	
Genetic islands	1 (38,915 bp)
CDS in genetic islands	53
CRISPR/Cas loci	Type II-C
Gene content/pathways	
Signal transduction	
Che proteins	7
Methyl-accepting chemotaxis proteins	12 [1]
Response regulators (RRs)	6
Histidine kinases (HKs)	5
Motility	
Flagellin genes	*flaA*
Restriction/modification	
Type I systems (*hsd*)	1
Type II systems	2 (type IIG, type IIP)
Type III systems	[1]
Type IV systems	*mcrBC*
Transcription/translation	
Transcriptional regulatory proteins	18 [1]
Sigma factors	σ^{28}, σ^{54}, σ^{70}
tRNAs; ribosomal loci	44; 3
Amino acid biosynthesis	
Arginine	No
Aromatic amino acids	No
Branched-chain amino acids	No
Histidine	No
Leucine	No
Lysine	Yes
Methionine, serine, cysteine	No
Proline	No
Catalase	*katA*
Cytolethal distending toxin (*cdtABC*)	Yes
Entner-Doudoroff pathway	Yes
Hippuricase	*hipO*
Leloir pathway	Yes
Nitrate/nitrite reductase	*napABDGH*, *nrfAH*
N-linked glycosylation (*pgl*)	Yes
Urease	No

[a] Numbers in square brackets indicate pseudogenes or fragments.
[b] Size listed in Mb due to length variation at the hypervariable G:C tracts.
[c] Numbers do not include pseudogenes; CDS, coding sequence.

Table S7: Comparison of the gene content of *C. molothri* and related taxa (the gene content profile for the *C. molothri* type strain is conserved within the other 28 *C. molothri* strains). Numbers in the *C. lari* group column indicate # of positive taxa if *n* < 8.

Cj jejuni^{T}	*Cj jejuni* NCTC 11168	*Cj doylei*^{T}	*C. coli*^{T}	*C. hepaticus*^{T}	*C. bilis*^{T}	*C. lari*^{T}	*C. lari* group (n=8)	*C. aviculae*^{T}	*C. estrildidarum*^{T}	*C. taeniopygiae*^{T}	*C. molothri*^{T}	Gene	Function
												**TCA cycle**	
✓	✓	✓	✓	✓	✓	✗	✗	✓	✓	✗	✗	***acnB***	aconitate hydratase 2
✓	✓	✓	✓	✓	✓	✓	✓	✓	✓	✓	✓	***frdA***	fumarate reductase, flavoprotein subunit
✓	✓	✓	✓	✓	✓	✓	✓	✓	✓	✓	✓	***frdB***	fumarate reductase, iron-sulfur subunit
✓	✓	✓	✓	✓	✓	✓	✓	✓	✓	✓	✓	***frdC***	fumarate reductase, cytochrome *b* subunit
✓	✓	✓	✓	✓	✓	✓	✓	✓	✓	✓	✓	***fumC***	fumarate hydratase, class II
✓	✓	✓	✓	✓	✓	✗	✗	✓	✓	✗	✗	***gltA***	citrate synthase
✓	✓	✓	✓	✓	✓	✗	✗	✓	✓	✗	✗	***icd***	isocitrate dehydrogenase, monomeric
✓	✓	✓	✓	✓	✓	✓	7	✓	✓	✓	✗	***mdh***	malate dehydrogenase, NAD-dependent
✓	✓	✓	✓	✓	✓	✓	✓	✓	✓	✓	✓	***mqo***	malate:quinone-oxidoreductase
✓	✓	✓	✓	✓	✓	✓	✓	✓	✓	✓	✓	***oorA***	2-oxoglutarate:acceptor oxidoreductase, alpha subunit
✓	✓	✓	✓	✓	✓	✓	✓	✓	✓	✓	✓	***oorB***	2-oxoglutarate:acceptor oxidoreductase, beta subunit
✓	✓	✓	✓	✓	✓	✓	✓	✓	✓	✓	✓	***oorC***	2-oxoglutarate:acceptor oxidoreductase, gamma subunit
✓	✓	✓	✓	✓	✓	✓	✓	✓	✓	✓	✓	***oorD***	2-oxoglutarate:acceptor oxidoreductase, delta subunit
✓	✓	✓	✓	✓	✓	✗	✗	✗	✗	✗	✗	***sucC***	succinyl-CoA synthetase, beta subunit
✓	✓	✓	✓	✓	✓	✗	✗	✗	✗	✗	✗	***sucD***	succinyl-CoA synthetase, alpha subunit
												**Arginine biosynthesis**	
✓	✓	✓	✓	✓	✓	✗	✗	✗	✗	✗	✗	***argB***	acetylglutamate kinase
✓	✓	✓	✓	✓	✓	✗	✗	✗	✗	✗	✗	***argC***	N-acetyl-gamma-glutamylphosphate reductase, common form
✓	✓	✓	✓	✓	✓	✗	✗	✗	✗	✗	✗	***argD***	N-succinyldiaminopimelate-aminotransferase / acetylornithine transaminase
✓	✓	✓	✓	✓	✓	✗	3	✗	✗	✗	✗	***argF***	ornithine carbamoyltransferase
✓	✓	✓	✓	✓	✓	✓	✓	✓	✓	✗	✗	***argG***	argininosuccinate synthase
✓	✓	✓	✓	✓	✓	✓	✓	✓	✓	✗	✗	***argH***	argininosuccinate lyase
✓	✓	✓	✓	✓	✓	✗	✗	✗	✗	✗	✗	***argO***	acetyltransferase
												**Serine / cysteine biosynthesis**	
✓	✓	✓	✓	✓	✓	✓	✓	✗	✗	✗	✗	***serA***	alpha-ketoglutarate reductase / D-3-phosphoglycerate dehydrogenase
✓	✓	✓	✓	✓	✓	✓	✓	✓	✓	✓	✓	***serB***	phosphoserine phosphatase
✓	✓	✓	✓	✓	✓	✓	✓	✓	✓	✓	✓	***serC***	phosphohydroxythreonine aminotransferase / 3-phosphoserine aminotransferase
✓	✓	✓	✓	✗	✗	✓	7	✗	✗	✗	✗	***cysE***	serine O-acetyltransferase
✓	✓	✓	✓	✗	✗	✓	7	✗	✗	✗	✗	***cysK***	cysteine synthase

Gene	Product	*Cj jejuni*^{T}	*Cj jejuni* NCTC 11168	*Cj doylei*^{T}	*C. coli*^{T}	*C. hepaticus*^{T}	*C. bilis*^{T}	*C. lari*^{T}	*C. lari* group (n=8)	*C. aviculae*^{T}	*C. estrildidarum*^{T}	*C. taeniopygiae*^{T}	*C. molothri*^{T}
**Lysine biosynthesis**													
asd	aspartate-semialdehyde dehydrogenase	✓	✓	✓	✓	✓	✓	✓	✓	✓	✓	✓	✓
dapA	4-hydroxy-tetrahydrodipicolinate synthase	✓	✓	✓	✓	✓	✓	✓	✓	✓	✓	✓	✓
dapB	4-hydroxy-tetrahydrodipicolinate reductase	✓	✓	✓	✓	✓	✓	✓	✓	✓	✓	✓	✓
dapD	tetrahydrodipicolinate succinylase	✓	✓	✓	✓	✓	✓	✓	✓	✓	✓	✓	✓
dapE	N-succinyl-diaminopimelate deacylase	✓	✓	✓	✓	✓	✓	✓	✓	✓	✓	✓	✓
dapF	diaminopimelate epimerase	✓	✓	✓	✓	✓	✓	✓	✓	✓	✓	✓	✓
lysA	diaminopimelate decarboxylase	✓	✓	✓	✓	✓	✓	✓	✓	✓	✓	✓	✓
lysC	aspartokinase, alpha and beta subunits	✓	✓	✓	✓	✓	✓	✓	✓	✓	✓	✓	✓
**Aromatic amino acid biosynthesis**													
aroA	3-phosphoshikimate 1-carboxyvinyltransferase	✓	✓	✓	✓	✓	✓	✓	✓	✓	✓	✓	✓
aroB	3-dehydroquinate synthase	✓	✓	✓	✓	✓	✓	✓	✓	✓	✓	✓	✓
aroC	chorismate synthase	✓	✓	✓	✓	✓	✓	✓	✓	✓	✓	✓	✓
aroD	3-dehydroquinate dehydratase	✓	✓	✓	✓	✓	✓	✓	✓	✓	✓	✓	✓
aroE	shikimate dehydrogenase	✓	✓	✓	✓	✓	✓	✓	✓	✓	✓	✓	✓
aroG	2-dehydro-3-deoxyphosphoheptonate aldolase (DAHP synthetase, class II)	✓	✓	✓	✓	✓	✓	✓	✓	✓	✓	✓	✓
aroK	shikimate kinase	✓	✓	✓	✓	✓	✓	✓	✓	✓	✓	✓	✓
pheA	chorismate mutase / prephenate dehydratase	✓	✓	✓	✓	✓	✓	✓	✓	✗	✗	✗	✗
trpA	tryptophan synthase, alpha subunit	✓	✓	✓	✓	✓	✓	✗	✗	✗	✗	✗	✗
trpB	tryptophan synthase, beta subunit	✓	✓	✓	✓	✓	✓	✓	✓	✗	✗	✗	✗
trpC	indole-3-glycerol phosphate synthase	✓	✓	✓	✓	✓	✓	✗	✗	✓	✓	✓	✓
trpDG	anthranilate phosphoribosyltransferase / anthranilate synthase component II	✓	✓	✓	✓	✓	✓	✗	✗	✗	✗	✗	✗
trpE	anthranilate synthase component I	✓	✓	✓	✓	✓	✓	✗	✗	✗	✗	✗	✗
trpF	phosphoribosylanthranilate isomerase	✓	✓	✓	✓	✓	✓	✗	✗	✗	✗	✗	✗
tyrA	chorismate mutase / prephenate dehydrogenase	✓	✓	✓	✓	✓	✓	✓	✓	✗	✗	✗	✗
**Glutamine/glutamate biosynthesis**													
gdhA	glutamate dehydrogenase	✗	✗	✗	✗	✗	✗	✓	5	✗	✗	✗	✗
glnA	glutamine synthetase	✓	✓	✓	✓	✓	✓	✓	✓	✓	✓	✓	✓
gltB	glutamate synthase, large subunit	✓	✓	✓	✓	✓	✓	✗	✗	✓	✓	✓	✓
gltD	glutamate synthase, small subunit	✓	✓	✓	✓	✓	✓	✗	✗	✗	✗	✗	✗

Gene	Cj jejuni[T]	Cj jejuni NCTC 11168	Cj doylei[T]	C. coli[T]	C. hepaticus[T]	C. bilis[T]	C. lari[T]	C. lari group (n=8)	C. aviculae[T]	C. estrildidarum[T]	C. taeniopygiae[T]	C. molothri[T]	
**Histidine biosynthesis**													
hisA	✓	✓	✓	✓	✓	✓	✓	✓	✗	✗	✗	✗	1-(5-phosphoribosyl)-5-[(5-phosphoribosylamino)methylideneamino]imidazole-4-carboxamide isomerase
hisBJ	✓	✓	✓	✓	✓	✓	✓	✓	✗	✗	✗	✗	imidazoleglycerol-phosphate dehydratase / histidinol-phosphatase
hisC	✓	✓	✓	✓	✓	✓	✓	✓	✗	✗	✗	✗	histidinol-phosphate aminotransferase
hisD	✓	✓	✓	✓	✓	✓	✓	✓	✗	✗	✗	✗	histidinal dehydrogenase / histidinol dehydrogenase
hisF	✓	✓	✓	✓	✓	✓	✓	✓	✗	✗	✗	✗	imidazole glycerol phosphate synthase HisFH, HisF subunit
hisG(L)	✓	✓	✓	✓	✓	✓	✓	✓	✗	✗	✗	✗	ATP phosphoribosyltransferase, hexameric long form
hisH	✓	✓	✓	✓	✓	✓	✓	✓	✗	✗	✗	✗	imidazole glycerol phosphate synthase HisFH, HisH subunit
hisIE	✓	✓	✓	✓	✓	✓	✓	✓	✗	✗	✗	✗	phosphoribosyl-AMP cyclohydrolase / phosphoribosyl-ATP pyrophosphatase
**Proline biosynthesis**													
proA	✓	✓	✗	✓	✗	✗	✗	✗	✗	✗	✗	✗	glutamate-5-semialdehyde dehydrogenase
proB	✓	✓	✗	✓	✗	✗	✗	✗	✗	✗	✗	✗	gamma-glutamyl kinase
proC	✓	✓	✓	✓	✓	✓	✓	✓	✗	✗	✗	✗	pyrroline-5-carboxylate reductase
**Branched-chain amino acid biosynthesis**													
ilvA	✓	✓	✓	✓	✓	✓	✓	✓	✗	✗	✗	✗	threonine deaminase
ilvC	✓	✓	✓	✓	✓	✓	✓	✓	✗	✗	✗	✗	acetohydroxy acid isomeroreductase
ilvD	✓	✓	✓	✓	✓	✓	✓	✓	✗	✗	✗	✗	dihydroxyacid dehydratase
ilvE	✓	✓	✓	✓	✓	✓	✓	✓	✓	✓	✗	✗	branched-chain amino-acid aminotransferase
ilvH	✓	✓	✓	✓	✓	✓	✓	✓	✗	✗	✗	✗	acetolactate synthase III, valine-sensitive, regulatory (small) subunit
ilvI	✓	✓	✓	✓	✓	✓	✓	✓	✗	✗	✗	✗	acetolactate synthase III, valine-sensitive, catalytic (large) subunit
leuA	✓	✓	✓	✓	✓	✓	✗	✗	✗	✗	✗	✗	2-isopropylmalate synthase, bacterial type
leuB	✓	✓	✓	✓	✓	✓	✗	✗	✗	✗	✗	✗	3-isopropylmalate dehydrogenase
leuC	✓	✓	✓	✓	✓	✓	✗	✗	✗	✗	✗	✗	isopropylmalate isomerase, large subunit
leuD	✓	✓	✓	✓	✓	✓	✗	✗	✗	✗	✗	✗	isopropylmalate isomerase, small subunit
**Methionine biosynthesis**													
metA	✗	✓	✓	✓	✓	✓	✗	✗	✗	✗	✗	✗	homoserine O-acetyltransferase
metC	✗	✗	✓	✓	✓	✓	✓	7	✓	✗	✓	✓	cystathionine-beta-lyase
metE	✓	✓	✓	✓	✓	✓	✗	✗	✗	✗	✗	✗	cobalamin-independent homocysteine transmethylase
metY	✗	✓	✓	✓	✓	✓	✗	✗	✗	✗	✗	✗	O-acetylhomoserine sulfhydrylase

Gene	Product	*Cj jejuni*[T]	*Cj jejuni* NCTC 11168	*Cj doylei*[T]	*C. coli*[T]	*C. hepaticus*[T]	*C. bilis*[T]	*C. lari*[T]	*C. lari* group (n=8)	*C. aviculae*[T]	*C. estrildidarum*[T]	*C. taeniopygiae*[T]	*C. molothri*[T]
**Pantothenate biosynthesis**													
panB	3-methyl-2-oxobutanoate hydroxymethyltransferase	✗	✓	✓	✓	✗	✗	✗	2	✗	✗	✗	✗
panC	pantothenate synthetase	✗	✓	✓	✓	✗	✗	✗	2	✗	✗	✗	✗
panD	aspartate 1-decarboxylase	✗	✓	✓	✓	✗	✗	✗	2	✗	✗	✗	✗
**Proline biosynthesis**													
proA	glutamate-5-semialdehyde dehydrogenase	✓	✓	✗	✓	✗	✗	✗	✗	✗	✗	✗	✗
proB	gamma-glutamyl kinase	✓	✓	✗	✓	✗	✗	✗	✗	✗	✗	✗	✗
proC	pyrroline-5-carboxylate reductase	✓	✓	✓	✓	✓	✓	✓	✓	✗	✗	✗	✗
**Entner-Doudoroff pathway**													
eda	multifunctional KHG/KDPG aldolase	✗	✗	✓	✗	✓	✓	✗	✗	✓	✓	✓	✓
edd	phosphogluconate dehydratase	✗	✗	✓	✗	✓	✓	✗	✗	✓	✓	✓	✓
glk	glucokinase	✗	✗	✓	✗	✓	✓	✗	✗	✓	✓	✓	✓
gluP	glucose/galactose transporter	✗	✗	✓	✗	✓	✓	✗	✗	✓	✓	✓	✓
pgiB	phosphoglucose isomerase (Entner-Doudoroff pathway-associated)	✗	✗	✓	✗	✓	✓	✗	✗	✓	✓	✓	✓
pgl	6-phosphogluconolactonase	✗	✗	✓	✗	✓	✓	✗	✗	✓	✓	✓	✓
zwf	glucose 6-phosphate 1-dehydrogenase	✗	✗	✓	✗	✓	✓	✗	✗	✓	✓	✓	✓
**Leloir pathway**													
galK	galactokinase	✗	✗	✗	✗	✗	✗	✗	✗	✓	✓	✓	✓
galM	galactose mutarotase	✗	✗	✗	✗	✗	✗	✗	✗	✓	✓	✓	✓
galT	galactose-1-phosphate uridylyltransferase	✗	✗	✗	✗	✗	✗	✗	✗	✓	✓	✓	✓
**Glycolysis**													
eno	enolase	✓	✓	✓	✓	✓	✓	✓	✓	✓	✓	✓	✓
fbaA	fructose-bisphosphate aldolase, class IIA	✓	✓	✓	✓	✓	✓	✓	✓	✓	✓	✓	✓
fbp	fructose-1,6-bisphosphatase I	✓	✓	✓	✓	✓	✓	✓	✓	✓	✓	✓	✓
gapA	glyceraldehyde 3-phosphate dehydrogenase A	✓	✓	✓	✓	✓	✓	✓	✓	✓	✓	✓	✓
pfk	phosphofructokinas	✗	✗	✗	✗	✗	✗	✗	✗	✗	✗	✗	✗
pgi	phosphoglucose isomerase	✓	✓	✓	✓	✓	✓	✓	✓	✓	✗	✓	✓
pgk	phosphoglycerate kinase	✓	✓	✓	✓	✓	✓	✓	✓	✓	✓	✓	✓
pgm	phosphoglycerate mutase	✓	✓	✓	✓	✓	✓	✓	✓	✓	✓	✓	✓
pykF	pyruvate kinase I	✓	✓	✓	✓	✓	✓	✓	✓	✓	✓	✓	✓

File S1: Phenotypic tests used in this study.

1) Oxidase activity.
   A nitrocellulose disk was saturated with 50 µL of Kovács oxidase reagent (1% (w/v) N,N,N',N'-Tetramethyl-p-phenylenediamine dihydrochloride in water) then dried. A 10 µl loop of bacteria was smeared across the disk. Dark blue color appearing within 15 sec indicated the presence of cytochrome oxidase.
   Control strains: (+) *C. jejuni* subsp. *jejuni* NCTC 11351^T
   (–) *C. suis* LMG 8286^T
   Reference: [1]

2) Catalase activity.
   Several drops of 3% (v/v) hydrogen peroxide were placed on a glass side, into which a 10 µl loop of bacteria was mixed. The presence of oxygen bubbles observed immediately after mixing bacteria indicated catalase activity.
   Control strains: (+) *C. jejuni* subsp. *jejuni* NCTC 11351^T
   (–) *C. helveticus* ATCC 51209^T
   Reference: [2]

3) Nitrate reduction.
   The Remel Nitrate Disk and reagent kit (Thermo Fisher, Waltham, MA) and protocol was used. A Nitrate disk was placed onto a heavily inoculated plate. The plate was incubated microaerobically at 37 °C for 24–48 h, after which the disk was removed to a clean surface. One drop each of Nitrate Reagents A and B was added onto the disk. Red color within 3–5 min indicated a positive result. If there was no color after 5 min, zinc powder was added, and the disk was observed for color change for an additional 5 min.
   Control strains: (+) *C. jejuni* subsp. *jejuni* NCTC 11351^T
   (–) *C. jejuni* subsp. *doylei* NCTC 11951^T

4) Indoxyl acetate hydrolysis.
   A nitrocellulose disk was saturated with 25 µL indoxyl acetate (10% (w/v) in acetone) and dried. A 10 µl loop of bacteria was placed onto the disk followed by a drop of distilled water. Dark blue color developing within 10 min was indicative of indoxyl acetate hydrolysis.
   Control strains: (+) *C. coli* ATCC 33559^T
   (–) *C. fetus* subsp. *fetus* NCTC 10842^T
   Reference: [2]

5) Urease activity.
   2 ml of a urea solution (3 mM sodium dihydrogen phosphate, 110 mM urea and 7 µg/ml phenol red, pH 6.8; modified Christensen urea) was dispensed into a test tube. A 10 µl loop of bacteria was resuspended in the urea solution, which was then incubated for 1 h at room temperature. A magenta color indicated the presence of urease.
   Control strains: (+) UPTC *C. lari* NCTC 11845
   (–) *C. fetus* subsp. *fetus* NCTC 10842^T
   Reference: [3]

6) Alkaline phosphatase activity.
Bacteria were resuspended densely into 0.5 ml of 0.2% p-nitrophenyl phosphate disodium tetrahydrate-0.1% glycine buffer containing 1 mM $MgCl_2$. After a 3 h incubation at 37 °C, the bacteria were spun down. The supernatant was transferred to a cuvette and a reading at 405 nm was made. Any yellow color above background was considered positive.
Control strains: (+) *C. lanienae* NCTC 13004^T
(–) *C. jejuni* subsp. *jejuni* NCTC 11351^T
Reference: [4]

7) Hippurate hydrolysis.
A 10 µl loop of bacteria was suspended in a microcentrifuge tube containing 200 µL 1% (w/v) sodium hippurate in water and incubated for 2 h at 37 °C. An equal volume of ninhydrin solution (3.5% (w/v) in 1:1 acetone: methanol) was then added and further incubated for 15 min at 37 °C. A dark purple color indicated hippurate hydrolysis.
Control strains: (+) *C. jejuni* subsp. *jejuni* NCTC 11351^T
(–) *C. coli* ATCC 33559^T
Reference: [5]

8) H_2S production on TSI agar.
Bacteria were inoculated onto a TSI agar slant (Thermo Fisher). The slant was incubated microaerobically in a hydrogen-containing atmosphere (1−2% O_2, 10% CO_2, 10% H_2, ~80% N_2) for 5 days at 37 °C. Blackening of the media indicated H_2S production.
Control strains: (+) *C. mucosalis* ATCC 43264^T
(–) *C. jejuni* subsp. *jejuni* NCTC 11351^T
Reference: [2]

9) α-hemolysis.
A 10 µL loop of actively growing bacteria was patched onto an Anaerobe Basal Agar plate supplemented with 5% lysed horse blood (ABA-B). The plate was incubated microaerobically at 37 °C for 48 h. The presence of a dark greenish-brown coloration around the perimeter of the patch was indicative of α-hemolysis.
Control strains: (+) *C. jejuni* subsp. *jejuni* NCTC 11351^T
(–) *C. mucosalis* ATCC 43264^T
Reference: [6]

10) Selenite reduction.
Bacteria were inoculated onto an ABA-B plate supplemented with 0.1% (w/v) sodium selenite and incubated at 37 °C for 48 h. The presence of orange color around the bacterial growth indicated selenite reduction.
Control strains: (+) *C. jejuni* subsp. *jejuni* NCTC 11351^T
(–) *C. helveticus* ATCC 51209^T

11) TTC reduction.
Bacteria were inoculated onto an ABA-B plate supplemented with 0.04% (w/w) 2,3,5-triphenyltetrazolium chloride. The plate was incubated microaerobically in a hydrogen-containing atmosphere (1−2% O_2, 10% CO_2, 10% H_2, ~80% N_2) for 48 h at 37 °C. A dark brownish-red color around the bacterial growth was indicative of TTC reduction.
Note: Some strains or species will not grow on media amended with 0.04% TTC. However, we have found that enough reductant is present within the initial patch/streak to produce red pigmentation. These results would indicate a growth$^-$/reduction$^+$ phenotype. Growth$^-$/reduction$^-$ phenotypes are also observed.
Control strains: (+) *C. coli* ATCC 33559^T
(–) *C. helveticus* ATCC 51209^T

12) Methyl orange.
Bacteria were inoculated onto ABA containing 0.032% (w/v) methyl orange and incubated at 37 °C for 48 h. Clearing of orange coloration indicates dye degradation.
Control strains: (+) *C. lari* subsp. *lari* LMG 8846^T
(–) *C. showae* ATCC 51146^T

13) Trimethylamine N-oxide.
Bacteria were inoculated onto ABA containing 0.1% (w/v) trimethylamine N-oxide and incubated anaerobically at 37 °C for 72 h. Absence of growth indicates inhibition.
Control strains: (+) *C. lari* subsp. *lari* LMG 8846^T
(–) *C. jejuni* subsp. *jejuni* NCTC 11351^T

References:

[1]: https://asm.org/getattachment/00ce8639-8e76-4acb-8591-0f7b22a347c6/oxidase-test-protocol-3229.pdf (accessed 6 Aug 2024).

[2]. On SL, Holmes B. Assessment of enzyme detection tests useful in identification of campylobacteria. *J Clin Microbiol* 1992;30:746-749.

[3] Owen RJ, Martin SR, Borman P. Rapid urea hydrolysis by gastric campylobacters. *Lancet* 1985; 1:111.

[4] Itoh T, Yanagawa Y, Shingaki M, Takahashi M, Kai A *et al*. Isolation of *Campylobacter pyloridis* from human gastric mucosa and characterization of the isolates. *Microbiol Immunol* 1987; 31:603-614.

[5] Skirrow MB, Benjamin J. Differentiation of enteropathogenic *Campylobacter*. *J Clin Pathol* 1980; 33:1122.

[6] Misawa N, Hirayama K, Itoh K, Takahashi E. Detection of alpha- and beta-hemolytic-like activity from *Campylobacter jejuni*. *J Clin Microbiol* 1995; 33:729-731.
